# Prognostic Value of Thyroid Hormone Ratio in Patients With Advanced Metastatic Renal Cell Carcinoma: Results From the Threefour Study (Meet-URO 14)

**DOI:** 10.3389/fonc.2021.787835

**Published:** 2021-11-25

**Authors:** Marco Maruzzo, Elena Verzoni, Maria Giuseppa Vitale, Michele Dionese, Sebastiano Buti, Luca Galli, Andrea Zivi, Sara Watutantrige-Fernando, Teresa Zielli, Elisa Zanardi, Roberto Sabbatini, Umberto Basso, Vittorina Zagonel, Giuseppe Procopio

**Affiliations:** ^1^ Oncology Unit 1, Istituto Oncologico Veneto, IOV - Istituto di Ricovero e Cura a Carattere Scientifico (IRCCS), Padova, Italy; ^2^ Genito-Urinary (GU) Oncology, Istituto di Ricovero e Cura a Carattere Scientifico (IRCCS) Fondazione Istituto Nazionale dei Tumori, Milano, Italy; ^3^ Oncology Unit, Azienda Ospedaliera Universitaria di Modena, Modena, Italy; ^4^ Dipartimento di Scienze Chirurgiche Oncologiche e Gastroenterologiche, Università degli Studi di Padova, Padova, Italy; ^5^ Oncology Unit, Azienda Ospedaliero-Universitaria di Parma, Parma, Italy; ^6^ Oncology Unit, Azienda Ospedaliera Universitaria Pisana, Pisa, Italy; ^7^ Oncology Unit, Azienda Ospedaliera Universitaria Integrata di Verona, Verona, Italy; ^8^ Hereditary Tumor Unit, Istituto Oncologico Veneto, IOV - Istituto di Ricovero e Cura a Carattere Scientifico (IRCCS), Padova, Italy; ^9^ Academic Unit of Medical Oncology, Istituto di Ricovero e Cura a Carattere Scientifico (IRCCS) San Martino Hospital, Genova, Italy

**Keywords:** renal cell carcinoma, FT3/FT4, deiodination, tyrosine kinase inhibitors, immunotherapy, progression, survival

## Abstract

**Background:**

Thyroid hormone impairment, represented as an alteration in levels of thyroid hormones and a lower fT3/fT4 ratio, has been correlated with a worse prognosis for both cancer and non-cancer patients. The role of baseline thyroid function in patients with metastatic renal cell carcinoma (mRCC) however, has not been studied yet.

**Materials and Methods:**

We recorded clinical data, baseline biochemical results, and oncological outcomes from 10 Oncology Units in Italy. We stratified patients into three groups according to the fT3/fT4 ratio value and subsequently analyzed differences in progression-free survival (PFS) and overall survival (OS) in the three groups. We also performed univariate and multivariate analyses to find prognostic factors for PFS and OS.

**Results:**

We analyzed 134 patients treated with systemic treatment for mRCC. Median PFS in the low, intermediate, and high fT3/fT4 ratio group were 7.5, 12.1, and 21.7 months respectively (p<0.001); median OS in the three groups were 36.5, 48.6, and 70.5 months respectively (p =0.006). The low fT3/fT4 ratio maintained its prognostic role at the multivariate analysis independently from IMDC and other well-established prognostic factors. The development of iatrogenic hypothyroidism was not associated with a better outcome.

**Conclusion:**

We found that baseline thyroid hormone impairment, represented by a low fT3/fT4 ratio, is a strong prognostic factor in patients treated for mRCC in first line setting and is independent of other parameters currently used in clinical practice.

## Introduction

In the last decades, the prognosis of patients affected with metastatic renal cell carcinoma (mRCC) progressively improved thanks to the development of new drugs that target tumor neoangiogenesis (tyrosine-kinase inhibitors of Vascular Endothelial Growth Factor Receptor, VEGFR) or promote the host immune response against tumoral cells (immune checkpoint inhibitors, ICI) (1, 2).

Despite the newest treatment options, however, some patients do not respond to systemic treatment or rapidly progress and die. Many prognostic scores were established over the years; the Memorial Sloan Kettering Cancer Center (MSKCC) and International Metastatic RCC Database Consortium (IMDC) risk score classification, the most commonly used in clinical practice, stratify patients into three risk groups taking into account clinical characteristics and biochemical examinations (3, 4). Those prognostic scores are currently used in daily clinical practice and clinical trials testing new drugs.

Thyroid hormone levels recently emerged as a prognostic factor in frail or elderly patients hospitalized for acute illness (5, 6). The presence of low levels of free triiodothyronine (fT3) in the absence of abnormalities in the thyroid function (defined as “euthyroid sick syndrome” or “non-thyroidal illness syndrome”, NTIS) has been shown as an independent prognostic factor for patients hospitalized for many different diseases (end-stage kidney disease, heart failure, acute coronary syndrome, etc.) (6–8). Levels of the active forms of thyroid hormones are due to deiodinases, a family of enzymes that can transform the biological precursor into the “active” or “inactive” forms (8).

In cancer patients, low fT3 levels have been correlated with a worse prognosis in patients affected by different solid malignancies (9–11).

The use of the fT3/fT4 ratio instead of the mean values of the two single hormones could be a better marker of peripheral deiodination activity and can even help stratify patients with normal fT3 levels. TSH is not usually used because its level usually remains within the normal range for several and different reasons (pituitary dysfunction, lower hypothalamic THR production, and reduced TSH pulsatility) and is thus less reliable (7).

Pasqualetti et al. found that a low fT3/fT4 ratio in non-cancer patients was strongly associated with frailty and was able to predict prognosis even in the case of normal fT3 levels in hospitalized elderly patients (6).

Recently, two papers showed that a low fT3/fT4 ratio predicts shorter overall survival (OS) and progression-free survival (PFS) in patients affected by metastatic colorectal cancer, independently of other established prognostic factors (12, 13).

In mRCC, the development of hypothyroidism during treatment with anti-VEGF tyrosine kinase inhibitors is a well-known favorable prognostic factor (14). However, the role of baseline thyroid values (and especially the fT3/fT4 ratio) has not been appropriately studied to date.

We, therefore, designed a multicentre retrospective trial to evaluate the correlation between the baseline fT3/fT4 ratio and outcomes of systemic treatments for mRCC.

## Material and Methods

The ThreeFour Study – Meet-URO 14 is a multicentre, retrospective, observational study. This study analyzed the clinical data of all consecutive mRCC patients treated from January 2007 to December 2014 at 10 Italian Oncology Units, within the Meet-URO cooperative group.

The study included patients aged 18 years or older with histologically confirmed mRCC who received first-line systemic treatment for metastatic disease and whose values of thyroid hormones were available. Patient data were collected retrospectively from clinical charts locally and imputed in a common, anonymized database. Demographic data, histological details (histological type, staging according to TMN, and grading), the risk group according to IMDC criteria, drugs used as first-line treatment, the values of thyroid hormones and the blood count were recorded for all patients, both at the baseline and at the time of the first radiological restaging, PFS and OS and best response to first-line treatment according to RECIST 1.1.

All the blood test was performed locally in the participant centres, in hospital certified laboratories. The ratio of fT3 and fT4 was calculated for each patient.

OS and PFS were evaluated with the Kaplan-Meier method from the start of the first-line treatment to the event of death for any cause or disease progression, respectively. All patients with no events were censored at the last follow-up. The OS and PFS in different groups were compared with the log-rank test and Cox’s proportional hazards method. Univariate and multivariate analyses were performed with a Wald test.

The study coordinated by the Istituto Oncologico Veneto (IOV) was approved by the Ethics Committee on 28 January 2019 and conducted according to the Declaration of Helsinki. Given the retrospective design and the fact that the majority of patients were dead at the time of analysis, the signed informed consent was not required from patients.

## Results

One hundred and ninety patients were included in the ThreeFour Study but only 134 had complete data available on thyroid hormones, since it is not a standard practice the baseline assessment of fT3 and fT4. Therefore, only patients with complete data and the possibility to calculate fT3/fT4 ratio were eligible for the analyses. Patients’ demographic data and principal clinical characteristics are reported in **Table 1**.

**Table 1:** Patient characteristics (N = 134).

Characteristics	Number of patients (%)
**Gender:**	
M	97 (72.4%)
F	37 (27.6%)
**Age (years)**	
Median (range)	63.4 (26.7-84.5)
>70 years (%)	44 (32.8%)
**Prior Nephrectomy**	
Yes	107 (79.9%)
No	27 (20.1%)
**Histology**	
Clear Cell	118 (88.1%)
Other histologies	16 (11.9%)
**Sarcomatoid features**	
Absent	130 (97.1%)
Present	4 (2.9%)
**Metastatic Sites**	
Lung	85 (63.4%)
Bone	17 (12.7%)
Liver	32 (23.9%)
CNS	5 (3.7%)
Lymph nodes	48 (35.8%)
Others	52 (38%)
**Number of metastatic sites**	
1	19 (14%)
2	62 (46%)
≥3	53 (40%)
**Baseline thyroid hormone levels**	
fT3 (median, range), pmol/l	3,81 (1,20-11,09)
fT4 (median, range), pmol/l	12,08 (4,04-21,10)
**IMDC risk classification**	
Good	38 (28.4%)
Intermediate	82 (61.2%)
Poor	13 (9.7%)
NA	1 (0.7%)
**Time from diagnosis to treatment**	
>12 months	62 (46.3%)
≤12 months	72 (53.7%)
**First-line treatment**	
TKI	105 (78.4%)
TKI + IT	15 (11.2%)
IT	14 (10.4%)

CNS, central nervous system; TKI, tyrosine kinase inhibitors; IT, immunotherapy; NA, not available.

The median age of the cohort was 63.4 years, with approximately one-third of patients older than 70 years. The vast majority of patients were affected by clear cell carcinoma with a prevalence of intermediate IMDC risk category (61.2%). The preferred option as first-line treatment was single-agent tyrosine kinase inhibitor (TKI) (sunitinib, pazopanib, tivozanib, lenvatinib plus everolimus; 78.4%) followed by a combination of immunotherapy and antiangiogenic therapy (11.2%) and immunotherapy alone (10.4%).

The best response reported by investigators during first-line treatment was stable disease in 62 cases (46%), partial response in 52 cases (39%), and disease progression in 16 cases (12%); complete response was only detected in 3 patients (2%).

One-hundred and six patients (79.1%) progressed and 62 (46.3%) died after a median follow-up of 29.4 months.

The median PFS and the median OS were 20.3 months (95% CI: 15-23.3 months) and 49.4 months (95% CI: 40.9 – 66.8 months) respectively, in the whole population.

The baseline fT3/fT4 ratio ranged from 0.13 to 4.87. In the whole cohort the higher fT3/fT4 ratio, considered as a continuous variable, was associated with better PFS (HR 0.819, 95% CI: 0.679-0.988) and OS (HR 0.672, 95% CI: 0.492-0.916) (**Table 2**).

**Table 2:** Univariate analysis of characteristics associated with PFS and OS.

Characteristics	PFS HR (95% CI)	p	OS HR (95% CI)	p
**Gender**				
F	–	–	–	–
M	0.874 (0.57-1.34)	0.536	0.598 (0.35-1.01)	0.0534
**Age (years)**	1.006 (0.99-1.02)	0.481	1.003 (0.98-1.01)	0.802
**Time from diagnosis to treatment**				
>12 months	–	–	–	–
≤12 months	1.661 (1.109-2.487)	**0.014**	1.461 (0.86-2.484)	0.161
**IMDC risk group**				
Good	–	–	–	–
Intermediate	1.579 (1.024-2.435)	**0.0389**	1.563 (0.868-2.814)	0.136
Poor	2.522 (1.189-5.347)	**0.0159**	3.617 (1.513-8.647)	**0.00384**
**Number of metastatic sites**	1.212 (0.967-1.519)	0.0943	1.252 (0.945-1.658)	0.118
**NLR**				
<3	–	–	–	–
≥3	1.511 (0.993-2.301)	0.054	1.933 (1.145-3.262)	**0.0136**
**fT3/fT4 ratio**	0.819 (0.679-0.988)	**0.0367**	0.672 (0.492-0.916)	**0.0119**
**fT3/fT4 ratio**				
low	–	–	–	–
intermediate	0.541 (0.333-0.877)	**0.0123**	0.640 (0.357-1.149)	0.135
high	0.427 (0.263-0.693)	**0.00057**	0.327 (0.171-0.624)	**0.000712**

The bold values are p significant values.

We subsequently stratified patients into three groups according to fT3/fT4 tertiles; 0.266 was the limit value between the low and intermediate group and 0.342 between the intermediate and high group.

**Figure 1:**
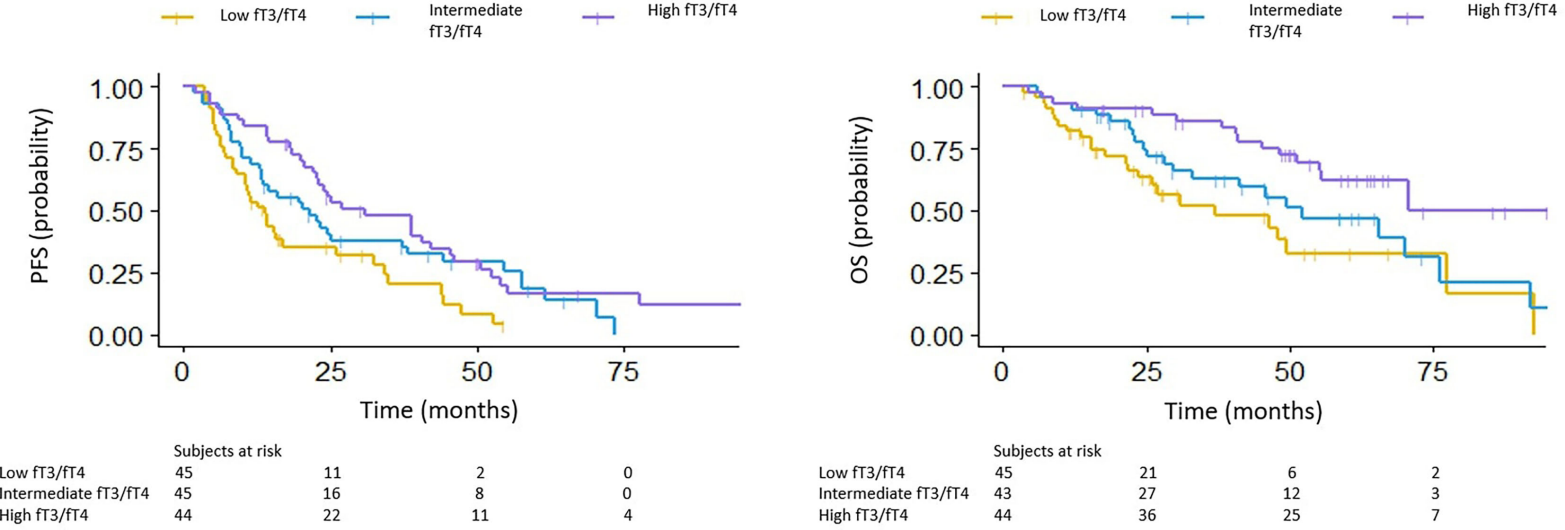
PFS (left) and OS (right) in the low, intermediate and high fT3/fT4 ratio groups.

The median PFS in the low, intermediate and high fT3/fT4 ratio group was 7.5, 12.1 and 21.7 months, respectively (p<0.001) (HR 0.54 for intermediate *vs* low fT3/fT4, 95% CI: 0.333-0.877; HR 0.43 for high *vs* low fT3/fT4, 95% CI: 0.263-0.693). The median OS in the low, intermediate and high fT3/fT4 groups was 6.5, 48.6 and 70.5 months, respectively (p =0.006) (HR 0.64 for intermediate *vs* low fT3/fT4, 95% CI: 0.357-1.149; HR 0.33 for high *vs* low fT3/fT4, 95% CI: 0.171-0.625) (**Figure 1**).

In the univariate analysis, the characteristics associated with PFS were the time from diagnosis to systemic treatment, the fT3/fT4 ratio, and the IMDC risk classification. A high neutrophil-to-lymphocyte ratio (NLR), a low fT3/fT4 ratio and, intermediate and poor risk according to the IMDC were associated with worse OS. Data on the type of treatment were not considered, given the heterogeneous variety of choice in the first-line treatment (**Table 2**).

In the multivariate analysis, the fT3/fT4 ratio was the only item that maintained a statistically significant association with PFS; a high fT3/fT4 ratio and a poor risk score based on the IMDC were factors associated with OS (**Table 3**).

**Table 3:** Multivariate analysis for PFS and OS.

Characteristic	HR for PFS (95% CI)	p	HR for OS (95% CI)	p
**Time from diagnosis to treatment**				
>12 months	–	–	–	–
≤12 months	1.351 (0.774-2.358)	0.289	0.923 (0.424-2.01)	0.840
**Number of sites**	1.005 (0.779-1.296)	0.967	1.026 (0.757-1.391)	0.867
**NLR**				
<3	–	–	–	–
≥3	1.061 (0.654 - 1.722)	0.812	1.461 (0.819- 2.604)	0.198
**fT3/fT4 ratio**				
low	–	–	–	–
intermediate	0.494 (0.281-0.867)	**0.0141**	0.524 (0.268-1.027)	0.0597
high	0.355 (0.199-0.635)	**0.0005**	0.293 (0.141-0.608)	**0.00097**
**IMDC risk group**				
Good	–	–	–	–
Intermediate	1.340 (0.745-2.413)	0.329	1.774 (0.762-4.130)	0.183
Poor	1.836 (0.821-4.05)	0.139	3.527 (1.291-9.632)	**0.0139**

The bold values are p significant values.

The disease control rate (represented by patients achieving stability, partial or complete response as the best response, DCR) was associated with the baseline fT3/fT4 ratio (DCR of 77.8%, 91.1% and 95.5% in the low, intermediate and high fT3/fT4 group, respectively; p=0.027).

During the treatment, the patients undergo thyroid function test at the time of radiological assessments; 69 of them developed clinical or subclinical hypothyroidism. PFS and OS did not significantly differ in those patients who developed hypothyroidism compared with patients without this complication (median PFS 11.9 *vs* 13.3 months, p NS; median OS 48.6 *vs* 54.9 months, p NS).

## Discussion

This study’s results cast new light on an interesting key connection of peripheral thyroid hormone metabolism with tumor progression and, thus, the survival of mRCC patients. The rationale of our analysis took inspiration from clinical situations that are different from cancer but with common features, such as cachexia or sarcopenia (15). Moreover, recent data were reported on the same topic for patients with colorectal carcinoma (12, 13).

The Italian cooperative group on urological oncology within the Meet-URO collected a robust dataset of patients diagnosed with mRCC and who were candidates to receive first-line treatment in accordance with clinical practice.

Our work shows that thyroid hormone dysfunction, represented by a lower fT3/fT4 ratio, is a strong prognostic factor for mRCC patients who underwent systemic treatment, confirming reported data for other neoplasms (11, 12).

The alteration of thyroid hormone values during acute or chronic illness (the so-called non-thyroidal illness syndrome [NTIS] which is not caused by an intrinsic dysfunction of the thyroid gland) is a common phenomenon that has been correlated with a worse prognosis in patients with active disease from many different causes (5–8).

It is currently unclear if those alterations represent a form of adaptive response to sickness or if those changes must be considered as real tissue hypothyroidism that needs to be corrected with hormone replacement therapy (8). The pathophysiology of NTIS takes into account various mechanisms ranging from alterations in the expression of the thyroid hormone receptor and thyroid hormone-binding protein, abnormal activity of the hypothalamic-pituitary axis (important in the initial phase of acute illness), and alteration in the thyroid hormone metabolism (8).

The levels of the active forms of thyroid hormones are in fact due to iodothyronine deiodinases, a family of enzymes that can transform the biological precursor T4 (produced by the thyroid) into the “active” form T3 (by deiodinases 1 and 2, or D1 and D2) or the inactive forms rT3 (from T4) and T2 (from T3) (by deiodinase 3, D3) (6, 8). The three deiodinases involved in the metabolic pathway, apart from their role, differ because of the tissue of expression: in particular, D1 is expressed in the liver and kidney and D2 in the skeletal muscle, where it is located within the cells and gives rise to most of the T3; D3 is considered an inactivating enzyme and it is important for placental and fetal tissues (8).

Chronic illness, cachexia, liver or renal impairment, and chronic systemic inflammation can lead to lower activity of D1 and D2 and overactivity of D3, thus leading to reduced levels of fT3 (16, 17). Those clinical situations are common in cancer patients, particularly in the end stages of the disease, and are typically associated with poor prognosis. Indirect markers of systemic inflammation (for example neutrophil and platelet count, NLR) are well-known negative prognostic factors (4, 18).

Therefore, NTIS and thyronine deiodinases impairment could be considered as an indirect marker of chronic systemic inflammation, cachexia, sarcopenia, or organ dysfunction, all characteristics that are associated with more advanced disease, a worse response to systemic therapy, and a worse prognosis.

However, the pathophysiological mechanism in cancer patients remains debatable, as does the role of the thyroid hormone and the activity of the deiodinases on cancer cell proliferation and differentiation (19). Besides, it is unclear if the administration of substitutive thyroid hormone therapy with triiodothyronine can lead to an improvement in the oncological outcome or, at least, in clinical symptoms, while it does not appear to be effective in non-cancer diseases (8, 16, 20).

In an oncological setting, many authors found a correlation between low fT3 levels and a worse prognosis in patients affected by advanced solid malignancies such as lung cancer and lymphomas (9, 11). Interestingly, elevated fT4 levels in hepatocarcinoma were correlated with a worse prognosis (10).

The use of the fT3/fT4 ratio was first proposed by Pasqualetti et al. as an indirect marker of deiodination impairment in their analysis of a cohort of hospitalized elderly patients (6). A low fT3/fT4 ratio correlated with frailty and worse survival, even in patients who had normal T3 values (6).

The fT3/fT4 ratio was studied in colorectal cancer patients, where it was identified as a strong prognostic factor in heavily pre-treated patients (12, 13). In their analysis, the prognostic role of the T3/T4 ratio was also independent of other well-known, established prognostic factors (12, 13).

In our cohort, contrary to what was previously reported (14), the development of treatment-induced hypothyroidism during treatment did not correlate with a better outcome in terms of PFS and OS (14). Apart from bias selection, one possible explanation could be the heterogeneity of treatment choices applied to patients followed in 10 different Institutions, where the development of hypothyroidism correlated with the outcome in patients treated with TKI monotherapy (14).

This study has many limitations, including the retrospective design with bias selection. Moreover, patients were restaged at different time points according to local practice. This probably led to a selection bias for patients with longer OS and PFS, thus resulting in survival curves that were far better than historical data. The sample size, albeit not small in absolute terms given the rarity of the disease, does not allow us to perform a subgroup analysis of the prognostic value of the fT3/fT4 ratio according to the type of systemic therapy (antiangiogenic, immunotherapy, or combination regimes) proposed. For the same reason, we could not test the predictive effect of the fT3/fT4 ratio in terms of response to different treatment options. Finally, the role of patients’ baseline iodine status and the possible presence of deiodinase polymorphisms were not studied in our cohort.

## Conclusion

In mRCC patients undergoing first-line systemic treatment, we identified the presence of baseline thyroid hormone impairment, quantified by a low fT3/fT4 ratio, as a strong prognostic factor for both PFS and OS. The role of the fT3/fT4 ratio in mRCC patients warrants validation in prospective cohorts to introduce it as a recognized prognostic factor for clinical practice as well as for clinical trials.

Moreover, additional studies are warranted to assess if the supplementation of thyroid hormones and correction of reduced deiodination of fT4 can improve the patient prognosis, and to also assess whether the changes in the levels of free thyroid hormones (binding proteins) play a role in cancer patients.

## Data Availability Statement

The raw data supporting the conclusions of this article will be made available by the authors, without undue reservation.

## Ethics Statement

The studies involving human participants were reviewed and approved by Comitato Etico dell’Istituto Oncologico Veneto. The patients/participants provided their written informed consent to participate in this study.

## Author Contributions

MM, conceptualization, methodology, formal analysis, supervision, and validation. EV, MV, SB, EZ, and RS, data curation, investigation, validation. MD, formal analysis, investigation, validation. LG, AZ, and TZ, data curation, investigation. SW-F, methodology, investigation, validation. UB, data curation, methodology, supervision, validation. VZ, supervision and validation. GP, methodology, supervision, validation. All authors contributed to the article and approved the submitted version.

## Conflict of Interest

The authors declare that the research was conducted in the absence of any commercial or financial relationships that could be construed as a potential conflict of interest.

## Publisher’s Note

All claims expressed in this article are solely those of the authors and do not necessarily represent those of their affiliated organizations, or those of the publisher, the editors and the reviewers. Any product that may be evaluated in this article, or claim that may be made by its manufacturer, is not guaranteed or endorsed by the publisher.
